# Drug sensitivity profiling and molecular characteristics of cells from pleural effusions of patients with lung adenocarcinoma

**DOI:** 10.18632/genesandcancer.56

**Published:** 2015-03

**Authors:** Rita Ötvös, Adam Szulkin, Carl-Olof Hillerdal, Aytekin Celep, Eviane Yousef-Fadhel, Henriette Skribek, Anders Hjerpe, László Székely, Katalin Dobra

**Affiliations:** ^1^ Karolinska Institutet, Department of Laboratory Medicine, Division of Pathology, Karolinska University Hospital, Stockholm, Sweden; ^2^ Karolinska Institutet, Department of Microbiology Tumor and Cell Biology (MTC), KI Solna Campus, Karolinska Institutet, Stockholm, Sweden

**Keywords:** Malignant pleural effusions, ex vivo chemo-sensitivity assay, RRM1, ERCC1

## Abstract

We propose to assess the therapeutic value of biomarker-guided individualized chemotherapy in patients with metastasizing lung adenocarcinoma. In this study, we used primary cells from pleural effusions from sixteen patients diagnosed with adenocarcinomas originating in the lung and from four patients with no malignant diagnosis. The *ex vivo* drug sensitivity of primary cells was assessed for 32 chemotherapeutical drugs. Linear regression analyses were performed to examine possible correlations between the drug sensitivity, overall survival and expression of ERCC1 and RRM1. The *ex vivo* drug sensitivity profiles of the patients revealed considerable heterogeneity in drug response. Vinblastine, vinorelbine, paclitaxel and actinomycin D showed high efficiency against 50% of the tested primary cells. Significant correlation was detected between the *ex vivo* sensitivity to platinum based drugs and gemcitabine and the level of ERCC1 and RRM1. No significant correlation was however seen between overall survival and drug sensitivity. The heterogeneity of the drug response suggests that optimal care of the adenocarcinoma patients should include the determination of drug sensitivity of the primary cells and would benefit to use personalized therapy.

## INTRODUCTION

Lung cancer is worldwide one of the leading causes of the cancer-related mortality for both men and women.[1] Non-small cell lung cancer (NSCLC) accounts for about 70 to 80% of all lung cancer cases. One subtype of NSCLC is the adenocarcinoma, which corresponds to about 40% of lung cancers.[2] Most cases of adenocarcinoma are associated with smoking; however it is also common in non-smokers and has become even more frequent in women than in men.[3] Primary lung adenocarcinoma are characterized by presence of thyroid transcription factor-1 (TTF-1).[4]

Accumulation of malignant pleural effusion (MPE) is common during NSCLC progression, particularly in adenocarcinomas.[5] The MPE has consistently been shown to indicate a poor prognosis, with a median survival time of 8 to 16 months after diagnosis.[6][7][8] It has been demonstrated that MPEs can be an excellent source for studying *ex vivo* and *in vivo* tumor progression and it reproduces the naturally occurring heterogeneity of these tumors.[9][10][11]

The principal chemotherapeutic agents used for treatment of lung adenocarcinoma are platinum analogues in combination with pemetrexed, taxanes or gemcitabine, but the response rates are only 30 to 40%.[12][13] This highlights the need of personalized cancer treatment for these patients. Nowadays an individualized therapy based on molecular biomarkers has been increasingly suggested. For example ribonucleotide reductase M1 (RRM1) and excision repair cross-complementation group 1 (ERCC1) status have been reported to correlate with the therapeutic efficiency of platinum drugs and gemcitabine.[14][15] [16] The RRM1 gene encodes the regulatory subunit of ribonucleotide reductase, an essential enzyme that catalyses the reduction of ribonucleoside diphosphates to deoxyribonucleotides which is required for the DNA synthesis. Increased level of RRM1 enables cells to more efficiently repair DNA damage, resulting in resistance to gemcitabine based therapy.[17][18] The function of the ERCC1 protein is predominantly the nucleotide excision repair of damaged DNA, a process that removes DNA adducts caused by platinum drugs.[19] ERCC1-negative tumors have a better response to adjuvant platinum-based chemotherapy, whereas high expression of ERCC1 results in drug resistance.[20]

In the present study we have investigated the drug sensitivity profile of exfoliated tumor cells from patients with metastatic lung adenocarcinoma in order to find successful chemotherapy agents that are not used in current treatment protocols. We attempt to correlate the expression levels of RRM1 and ERCC1 to sensitivity patterns and survival time of patients. We also calculated the drug efficiency by adjusting for drug effect on benign cells and proportion of tumor cells.

## RESULTS

### Drug sensitivity patterns of primary cells

**Figure 1:**
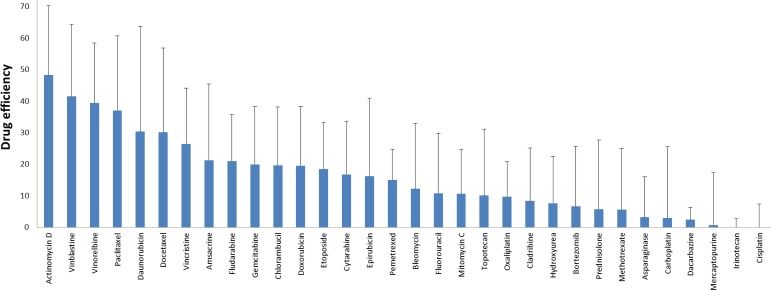
Summarized drug effect values of twenty-three adenocarcinoma cell isolates for 32 different cytostatic drugs The killing effects of the drugs were given on a 0-100 scale. No cell killing effect of the drugs gives 0, and killing all the cells even at the lowest concentration gives 100. Bars represent the standard deviation of the summarized drug effect.

We have evaluated the drug sensitivity patterns of primary cells derived from pleural effusions of sixteen patients diagnosed with lung adenocarcinoma and from pleural effusions of four patients with no malignant diagnosis, using an *ex vivo* sensitivity assay. In order to avoid changes in the composition of the cell cultures, all samples were tested within 3 days. Each sample was tested against 32 different drugs and in each well the percentage of surviving cells was calculated after 72 hours of treatment. The averaged drug effect is shown in Figure 1. Most of the primary cells were sensitive to actinomycin D, vinblastine, vinorelbine and paclitaxel with a drug effect of 37 to 48. At the same time most primary cells were resistant to oxaliplatin, cladribine, hydroxyurea, bortezomib, prednisolone, methotrexate, asparaginase, carboplatin, dacarbazine, mercaptopurine, irinotecan and cisplatin, where killing effect was <10. Six of cell cultures were treated with a slightly lower concentration of actinomycin D (fifth of the highest concentration), but except for one case, at the highest concentration did not affect 50% of the cells, the drug effect ranging from 19 to 28 (data not shown).

### Heat map of cluster analyses

**Figure 2:**
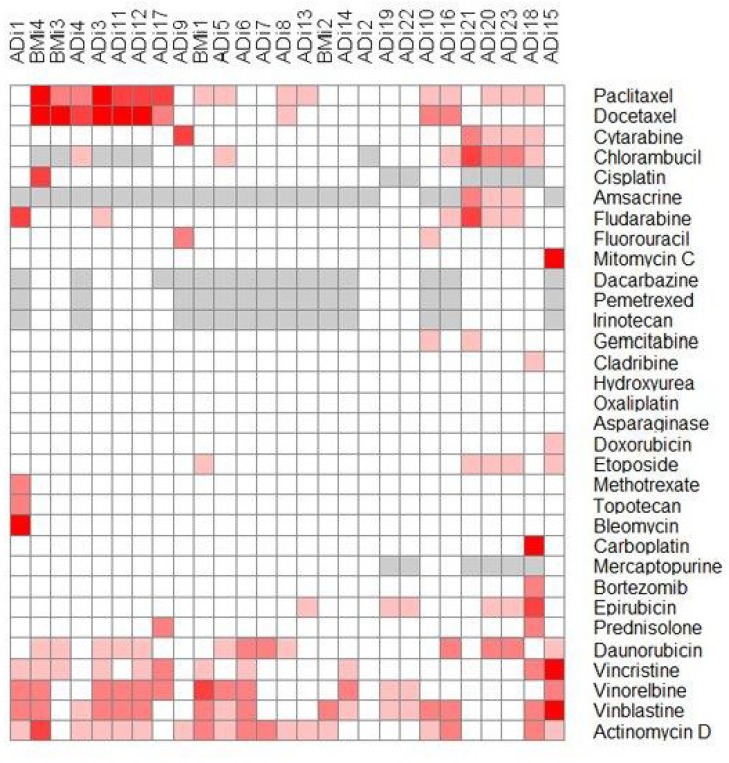
Heat map representation of the hierarchical clustering of drug sensitivity data of the individual primary cells against all drugs Intensity of the red color shows the sensitivity. White represents resistant and the intensity of the red color are proportional to the effect of the drug. Grey color means that the drug has not to be tested on this sample ADi1-23: adenocarcinoma cell isolates, BMi1-4: benign cell isolates.

In order to identify possible co-segregation of the sensitivity patterns of the individual drugs and to systematically compare all primary cells with each other, we used Euclidean distance metrics for two-dimensional hierarchical clustering of the drug sensitivity data. The sensitivity of the drug was represented on a five step scale where every step represents less than 50% viability at the four different drug dilutions. Cell isolates with more than 50% live cells were defined as resistant and were presented in white, while experiments with less than 50% of the cells alive at any tested concentration were considered sensitive and were presented in red. Increasing red colors represent sensitivity at lower drug concentrations. Grey means not tested. The graphical presentation of the clustering is shown in Figure 2.

Large differences in the chemo-sensitivity of the different primary cell isolates were observed. The proportion of malignant cells in the cell isolates tested for chemo-sensitivity ranged between 10 and 70%. The survival time of the patient from whom the samples were received ranged between 0 and 37 months after diagnoses. Cell isolates from patients with longer survival time seem to be less sensitive to the different drugs and have a slightly higher proportion of malignant cells (Figure 2).

ADi2 was the most resistant cell isolate, not affected by any of the tested drugs. ADi7 was affected by two of the drugs, while several of the isolates were affected by three of the drugs (ADi9, ADi14, ADi22, ADi13 and ADi19). The most sensitive cell isolate, ADi18 was affected by eleven drugs. Multiple isolates were affected by eight different drugs (ADi23, ADi3, ADi1, ADi15 and ADi20). The benign cell isolates were affected by between two and eight drugs (BMi1, BMi2, BMi3 and BMi4).

Actinomycin D, vinblastine, vinorelbine, paclitaxel and daunorubicin were the most effective drugs and affected between 13 and 17 of the malignant cell samples, but similar effect was also seen on the benign cell isolates. Docetaxel, chlorambucil, fludarabine, and epirubicin affected between 6 and 10 of the malignant cell samples while the benign isolates were less affected by these drugs. Several of the tested drugs did not affect any of the primary isolates and this was the case for drugs conventionally used to treat lung adenocarcinoma patients.

### Longer survival in patients with samples sensitive to less drugs

**Figure 3:**
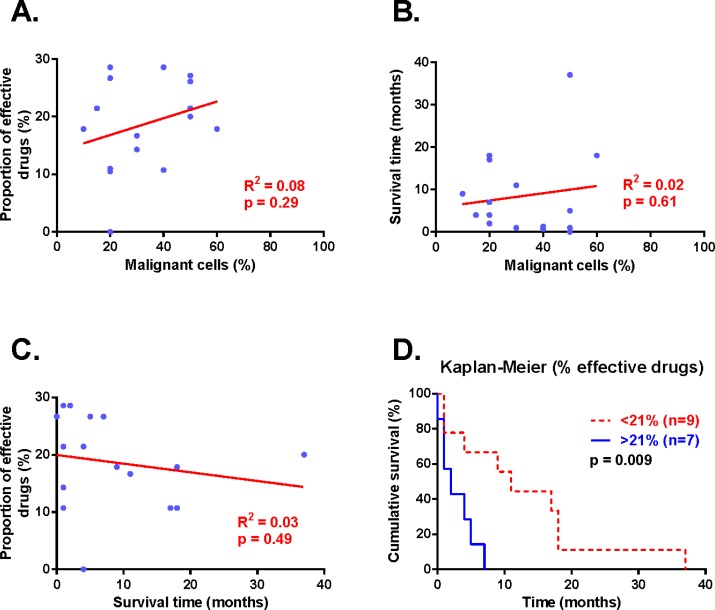
Correlation between proportion of effective drugs, percent malignant cells and survival time Correlations of the proportion of effective drugs, survival time and percent of malignant cells plotted in A-C. Each data point in blue represents one patient, result from the linear regression analyses are presented in red. Kaplan-Meier analysis presents survival time according to proportion of effective drugs (D). Statistical significance was accepted at p<0.05 and was seen for proportion of effective drugs and survival time in the Kaplan-Meier analysis.

In the survival analysis we included the original sample (ADi6, ADi11 and ADi15) and the papillary groups resembling the clinical manifestation of tumor (ADi20 and ADi23). The proportion of malignant cells was not correlated to the proportion of effective drugs or the survival time of patients in the linear regression analyses (Figure 3A and B). Similarly, survival time and proportion of effective drugs was not correlated (Figure 3C). But Kaplan-Meier analysis showed a significantly longer survival time for patients with cell isolates affected by less than 21% of the drugs compared to patients with samples affected by more than 21% of the drugs (p=0.009, figure 3D).

### Adjusting drug effect increased the amount of affected cell isolates

**Figure 4:**
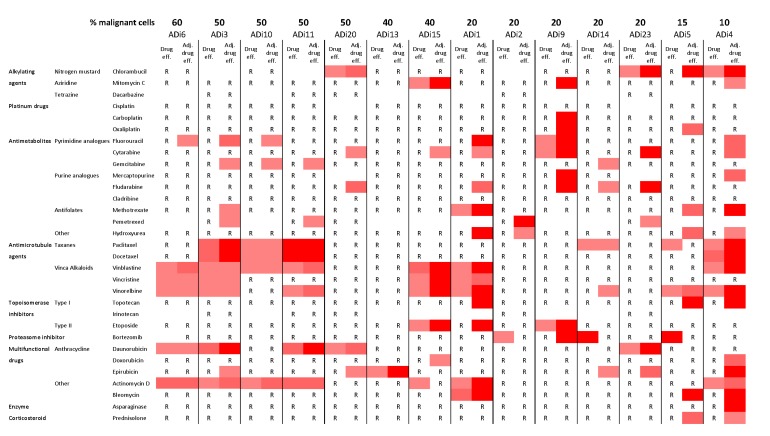
Drug efficiency and adjusted drug efficiency of lung adenocarcinoma cells 14 malignant cell isolates arranged according to their proportion of malignant cells. R = Resistant, drug efficiency less than 50%. An increasing red color represents increased drug efficiency (50-60%, 60-80% and 80-100%, respectively). Adjusting the drug efficiency for proportion of malignant cells and benign effect seems to increase the sensitivity of cell isolates and effects of several drugs.

The drug effect on 14 cell isolates was studied and adjusted for average effect on benign cells and proportion of malignant cells in each sample (Figure 4). This adjustment increased the drug effect in almost all samples, independent of the proportion of malignant cells. Several of the cell isolates however remained resistant to a majority of tested drugs. The adjustment also highlighted the potency of some drugs, not only did the effect of pemetrexed increased, but also the effect of fluorouracil, cytarabine, fludarabine, vinblastine, vinorelbine and actinomycin D. These drugs showed efficiency in 36 to 43% of the cells in the primary cell cultures.

### Immunocytochemical assessment of ERCC1 and RRM1

**Figure 5:**
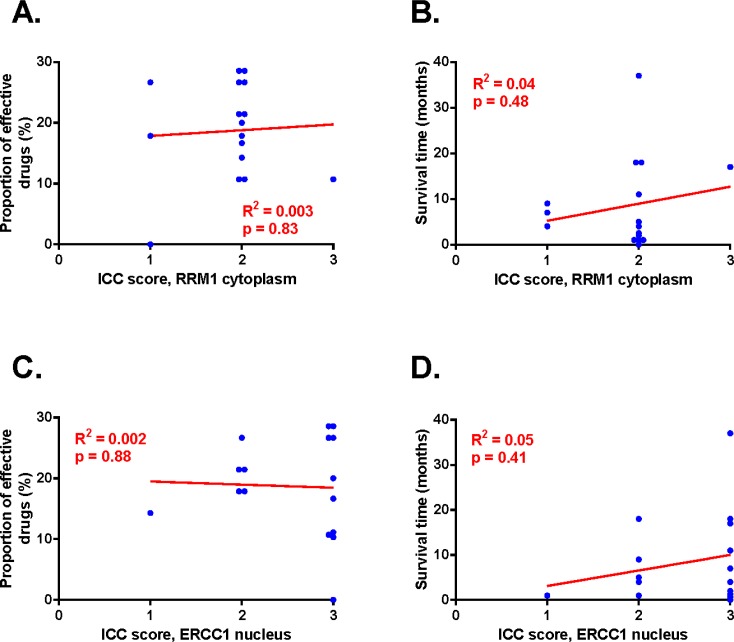
RRM1 and ERCC1 immunoreactivity correlated to proportion of effective drugs and survival time A: Proportion of effective drugs plotted against RRM1 staining. B: Survival time plotted against RRM1. C: Proportion of effective drugs plotted against ERCC1 staining. D: Survival time plotted against ERCC1. Regression analyses presented in red, data points for each patient in blue. Statistically significant departure of the slope from 0 was accepted at p<0.05 and was not seen.

The RRM1 staining was localized and evaluated to the cytoplasm and the ERCC1 staining was localized to the cell nucleus (Additional file 3.). The staining intensities for both proteins were graded on a scale from 0 to 3 corresponding to none (0), low (1), moderate (2) and strong (3) staining intensity. There was no significant difference between high or low expression of either RRM1 or ERCC1 and any of the clinical variables analyzed, which included age and gender (Figure 2). The expression level of RRM1 and ERCC1 showed no statistically significant correlation to survival time or to the proportion of effective drugs (Figure 5).

All four recurrent samples show decreased sensitivity against the tested drugs. Statistically significant correlation was observed between the cytotoxicity of carboplatin/cisplatin and gemcitabine and the levels of RRM1 and ERCC1 expression (Figure 2).

### Subgroup analysis

We performed several comparisons dividing the 16 samples in two equal groups, according to high or low proportion of malignant cells, long or short survival time and high or low proportion of effective drugs. For all these analyses, no significant differences was found using a two-tailed unpaired t-test, comparing the different parameters in the two groups (data not shown). When only looking at the seven cell isolates with the highest proportion of malignant cells (above 39% malignant cells) or only on the ten cell isolates with the strongest nuclear ERCC1 staining, no significant correlations were found by linear regression analysis between the different measured factors (data not shown).

All adenocarcinoma showed strong staining for TTF-1, except for two: ADi10 and ADi13 were negative for TTF-1. These MPEs were received from patients who were previously diagnosed with lung cancer. In these cell isolates, the proportion of malignant cells were higher than average (40 and 50%), the RRM1 and ERCC1 staining was similar but the survival time was shorter, only one month. ADi10 was affected by six drugs and ADi13 by three drugs.

### Evaluation of cell isolates originating from the same patient

The importance of how primary cells were isolated and the properties of the different fractions were studied. Five of the pleural effusions were separated in different ways (Table 1.) in order to investigate different fractions of the MPEs.

**Table 1:** Patients' characteristics

Culture identity	Age (years)	Gender	Cell culture condition	Survival (months)	Immunocytochemical staining		
					ERCC1	RRM1	TTF-1
ADi1	75	F	Primary cell culture	2	3	2	+
ADi2	82	F	Primary cell culture	4	3	1	+
ADi3	63	F	Primary cell culture	5	2	2	+
ADi4	50	F	Primary cell culture	9	2	1	+
ADi5	89	F	Primary cell culture	4	2	2	+
ADi6*	57	M	Primary cell culture	18	2	2	+
ADi7*			Spin down before seeding		3	2	
ADi8	67	F	Primary cell culture	1	1	2	+
ADi9	68	M	Primary cell culture	18	3	2	+
ADi10	64	F	Primary cell culture	1	2	2	−
ADi11*	74	M	Primary cell culture	37	3	2	+
ADi12*			Seeded after four days		2	2	
ADi13	80	M	Primary cell culture	1	3	2	−
ADi14	85	F	Primary cell culture	17	3	2	+
ADi15*	77	F	Primary cell culture	1	3	2	+
ADi16*			Seeded the following day		2	2	
ADi17	65	F	Primary cell culture	11	3	2	+
ADi18*	67	F	Adhered dissociated cells	0	3	3	+
ADi19*			Adhered papillary groups		3	3	
ADi20*			Floating papillary groups		3	3	
ADi21*	64	M	Adhered dissociated cells	7	2	2	+
ADi22*			Adhered papillary groups		1	2	
ADi23*			Floating papillary groups		3	2	

* Cell culture derived from the same patient

Two cell isolates were seeded two or four days after receiving the samples. This increased the proportion of malignant cells somewhat and the isolates showed distinctly altered pattern of drug sensitivity.

Two of the MPEs were seeded and then divided into three different cell cultures, adherent disassociated cells, adherent papillary groups and floating papillary groups. A comparison of the three different subgroups indicates that the adherent papillary groups are less sensitive to the tested drugs, compared to the adherent dissociated and floating papillary groups. The floating papillary groups showed a lower nuclear RRM1 staining.

## DISCUSSION

Lung cancer is one of the tumor types with the highest incidence and mortality worldwide. Chemotherapy is the most common therapy for patients with advanced lung cancer, only a minority of patients having resectable tumors.[13] Currently, therapy with a platinum drug combined with a second drug is the standard first-line treatment, with response rates of 30 to 40%.[13] Better understanding of the biological characteristics of the tumor and predicting response to chemotherapy is needed to improve the clinical outcome of patients with advanced adenocarcinomas.[17] The admixture of benign cells may obscure the results, and is one reason for previous poor correlation between *in vitro* sensitivity and patient response. The obtained variability in drug response is, however, striking, also when correcting for the content of benign cells. The present study shows that malignant pleural effusions obtained from lung cancer patients can be used to study chemo-sensitivity of adenocarcinoma.

Despite that the primary cells are received from different patients, they show a remarkably similar sensitivity pattern for a number of drugs (paclitaxel, vinblastine, vinorelbine, actinomycin D), but overall a considerable heterogeneity was seen regarding the drug sensitivity. Only one sample was highly resistant to most of the drugs whereas six samples showed good overall sensitivity, with effects from 25% or more of the drugs. Clustering of the drug sensitivity data revealed that the profiles are independent of the age, gender, survival time and expression of RRM1, ERCC1 and TTF-1. Surprisingly, cell isolates from patients with longer survival time seem to be less sensitive to the different drugs and have a slightly higher proportion of malignant cells. One hypothesis might be that tumors with slower proliferation react less to drugs with effects on just proliferation. Inversely, those isolates with less response would grow more slowly, hence with longer survival.

Pemetrexed has been approved as first- and second-line treatment in patients with NSCLC and belongs to antimetabolites, exerting a cell-cycle specific effect. We and others have shown that it has an antiproliferativ effect by inducing cell cycle arrest and S phase accumulation. [11] Therefore the drug affects dividing cells, by inhibiting several key enzymes in the folate-dependent metabolism and causing a decreased nucleotide synthesis. Effect of pemetrexed was observed when adjusting the efficiency for the proportion of malignant cells and average effect on benign cells.

Several examples of individualized therapy based on molecular and immunocytochemical analysis of biomarkers in pleural effusion have been published.[17][21][22][23] In this study we integrated immunocytochemistry with the *ex vivo* chemo-sensitivity assay. Our results show a significant correlation between level of ERCC1 and RRM1 with the effect of platinum agents and gemcitabine. No significant correlation was seen between overall survival and drug sensitivity in patients with adenocarcinoma. Low levels of RRM1 did not predict better outcomes and ERCC1 expression did not have a prognostic value in this patient cohort. Our findings are in harmony with previous results reported by Valsecchi et al. but opposite to results from Akita et al. that found better outcome for patients with high expression of RRM1 and ERCC1.[24][25] Immunocytochemistry (ICC) provides information on the amount of protein in the cell and its localization; however the commonly used ERCC1 antibody, mAb8F1[26][ 27], is known to give un-specific cytoplasmic staining and unspecific binding to unknown protein with a similar size. Thus, further studies are needed to establish the role of ERCC1 in metastasizing lung adenocarcinomas.

We were able to subculture two primary cell isolates adherently and in suspension.[9] Surprisingly drugs with similar mechanism of action varied in their effects on samples in different conditions from the same patient. Floating papillary groups had a higher proportion of malignant cells and were more sensitive than the adhered papillary groups. This deserves further analysis and experiments to get a thorough understanding. Meanwhile, it is important to mention that our study has its limitations in the number of patients.

In summary, the analyses of drug sensitivity profiles of the adenocarcinoma patients revealed considerable heterogeneity in drug effect. Vinblastine, vinorelbine, paclitaxel and actinomycin D showed higher efficiency against at least 50% of the tested primary cells. These drugs are not included in the current chemotherapy protocols for treatment of adenocarcinoma but are interesting to further evaluate. However, the heterogeneity of the drug effect also suggest that optimal care of the adenocarcinoma patients would include determination of drug sensitivity and would benefit from the use of personalized therapy. In order to validate pharmacogenetic and different candidate biomarkers for adenocarcinoma in clinical settings, further studies using multiple approaches are warranted.

## MATERIALS AND METHODS

### Patients characteristics and inclusion criteria

In this study, we used 23 cell isolates from pleural effusion of 16 patients diagnosed with metastatic adenocarcinoma originating in the lung and pleural effusions from four patients with benign pleural effusions. The malignant samples were received from five men and eleven women with a median age of 67.5 years, ranging from 50 to 89 years. The effusions were received from the diagnostic routine at the Department of Pathology and Cytology, Karolinska University Hospital in Huddinge, Sweden. The samples were collected between 2008 and 2011 and the study was approved by the regional ethics committee in Stockholm. The baseline clinico-pathologic characteristics of the patients are summarized in Table 1.

Malignant cells obtained from pleural effusions were evaluated through a combination of cytomorphology and ICC in order to confirm their phenotype and origin. The immunocytochemical analysis comprised of a staining profile including the epithelial cell adhesion molecule BerEp4, CK7, TTF-1 and Napsin A supporting an alien cell population of lung origin. The proportion of malignant cells was estimated from two cytospin preparations, one stained by BerEp4 immunocytochemistry and one according to MGG. The proportions were evaluated independently by two experienced cytopathologists (KD and AH), discussing discrepant cases to reach consensus.

The included benign pleural effusions contained benign mesothelial cells with admixture of inflammatory cells, without further information of their etiology and without any morphological sign of malignancy. Six months after collecting the benign effusions all four patients were still alive without any diagnosed malignancy. These cell isolates have previously been used as control cells in similar experiments with mesothelioma cells.[10]

### Cell culturing

For culturing of primary cells the effusions were centrifuged at 400 g, 5 min and cells were seeded in Iscove's modified Dulbecco's medium (Sigma-Aldrich, St. Louis, USA) containing 20% FBS (Fetal Bovine Serum, Invitrogen, Carlsbad, USA), 0.2% Gentamicin (Invitrogen), 1% Penicillin Streptomycin (Invitrogen) and 1% L-glutamine (Invitrogen). To optimize the cell isolation procedure, the appearance of the tumor cells in the effusion was considered. Thus two effusions contained substantial amounts of papillary groups; these groups were separated from disassociated cells by shaking the cell flasks after 24 hours, collecting the floating cells and reseeding them in new flasks. These cell isolates were then separated a second time by reseeding the floating papillary groups (summarized in Table 1).

### Cytotoxicity assay

Adherent primary cells were grown to 80-95% confluence, washed with phosphate buffer saline and trypsinized (Invitrogen). They were than counted and seeded. To test the *ex vivo* drug sensitivity of primary cells, the iVV assay (QantaScope AB, Sweden) was used, where the primary cells were assessed using a 3-day culture on 384 well microtiter plate as previously described.[28] [29] Briefly, 32 drugs (Additional file 1) were tested, each at four concentrations (diluted 1:1, 1:5, 1:25 and 1:125) covering a clinically relevant concentration span. Each well was loaded with 30μl OmniSanguine (QantaScope AB, Sweden) primary cell culture medium containing 3.000-5.000 cells.[30] Untreated control cells were grown on the same plate and in the same conditions. After 72 hours of incubation the living and dead cells were differentially stained using VitalDye (QantaScope AB, Sweden). The precise numbers of living and dead cells were measured using Qantascope HexascopeHAEMA automated scanning and analyzing system (Qantascope AB, Stockholm, Sweden).

For each sample the drug effects were automatically calculated as previously described.[10] Briefly, the drug effect is given on a 0-100 scale where no cell killing effect of the drugs gives 0, and killing all the cells even at the lowest concentration gives 100. A considerable variation in the proportion of tumor cells in the different cell isolates was observed. This was compensated by correcting the drug efficiency to the proportion of tumor cells and the supposed effect of drugs on the benign cells present in the preparation, the resulting measure being called “adjusted drug efficiency”.[10] The latter effect was estimated assuming that the benign cells present were affected by the individual drugs in the same magnitude as the corresponding average from the four benign control samples.

### Immunocytochemistry

Cytospin preparations of adenocarcinoma cells were performed on SuperFrost Plus glass slide (Thermo Fisher Scientific Inc., Waltham, MA, USA), fixed with 25% ethanol, 25% methanol, 3% polyethylene glycol (PEG) in H_2_ O and stored at −20°C. PEG was extracted before staining, by decreasing concentrations of ethanol. Immunostaining was performed in a Leica BOND-III automated IHC with relevant isotype controls, diluted in BOND Primary Antibody Diluent (Leica Microsystems GmbH) and detected with the Bond Polymer Refine Detection kit (Leica Microsystems GmbH) or Bond Polymer Refine Red Detection kit (Leica Microsystems GmbH) according to the manufacturer's protocol. Briefly, for detection of BerEp4, Calretinin, TTF-1, Napsin A and CD45 slides were pretreated 5 min in a citrate buffer pH 6.0 (Bond Epitope Retrieval Solution 1, Leica Microsystems GmbH), while an EDTA buffer pH 9.0 (Bond Epitope Retrieval Solution 2, Leica Microsystems GmbH) was used for 20 min for ERCC1 and RRM1 staining. Endogenous peroxidase activity was abolished with 3% hydrogen-peroxide. Slides were then treated with primary antibodies according to Additional file 2 for 30 min whereafter secondary antibody carrying poly-HRP was added and incubated for 15 min. Bound antibodies were then visualized by Diaminobenzidine treatment for 10 min and followed by 10 min counterstain with hematoxylin. Double staining was performed for Ber-EP4 and Calretinin as well as TTF-1 and Napsin A, to distinguish Ber-EP4 and TTF-1 positive adenocarcinoma cells from mesothelial cells. Ber-EP4 and TTF-1 were detected as described above whereafter Calretinin and Napsin A were detected with a secondary conjugated antibody, incubated with poly-AP and developed with Fast red. All slides were independently evaluated by two experienced cytopathologists (KD and AH) who rated the amount of malignant cells from 0-100% and the staining intensity from 0 to 3 (0 representing no staining and 3 representing strong staining). Discrepant cases were re-evaluated and discussed to reach consensus.

### Statistical analysis

Linear regression analyses were performed to examine possible correlations. A Log-rank (Mantel-Cox) test with two-tailed p-values was performed to compare survival data in the Kaplan-Meier plots. Subgroup comparisons were performed with an unpaired t-test with two-tailed p-values. The association between the level of ERCC1 and RRM1 and chemo-sensitivity of the primary cells was calculated using a linear regression analysis as well. For the two-dimensional hierarchical clustering the GENE-E 3.0.210 program (Broad Institute) was used.

## SUPPLEMENTARY MATERIAL TABLES AND FIGURE



## References

[1] Ferlay J, Shin HR, Bray F, Forman D, Mathers C, Parkin DM (2010). Estimates of worldwide burden of cancer in 2008: GLOBOCAN 2008. Int J Cancer.

[2] Sakashita S, Sakashita M, Sound Tsao M (2014). Genes and pathology of non-small cell lung carcinoma. Semin Oncol.

[3] Samet JM, Avila-Tang E, Boffetta P, Hannan LM, Olivo-Marston S, Thun MJ, Rudin CM (2009). Lung cancer in never smokers: clinical epidemiology and environmental risk factors. Clin Cancer Res.

[4] Castro CY, Moran CA, Flieder DG, Suster S (2001). Primary signet ring cell adenocarcinomas of the lung: a clinicopathological study of 15 cases. Histopathology.

[5] Han HS, Yun J, Lim SN, Han JH, Lee KH, Kim ST, Kang MH, Son SM, Lee YM, Choi SY, Yun SY, Kim WJ, Lee OJ (2013). Downregulation of cell-free miR-198 as a diagnostic biomarker for lung adenocarcinoma-associated malignant pleural effusion. Int J Cancer.

[6] Masters GA, Argiris AE, Hahn EA, Beck JT, Rausch PG, Ye Z, Monberg MJ, Bloss LP, Curiel RE, Obasaju CK (2006). A randomized phase II trial using two different treatment schedules of gemcitabine and carboplatin in patients with advanced non-small-cell lung cancer. J Thorac Oncol.

[7] Zhang H, Liu HB, Yuan DM, Wang ZF, Wang YF, Song Y (2014). Prognostic value of secreted phosphoprotein-1 in pleural effusion associated with non-small cell lung cancer. BMC Cancer.

[8] Bell D, Wright G (2013). A retrospective review of the palliative surgical management of malignant pleural effusions. BMJ Support Palliat Care.

[9] Mancini R, Giarnieri E, De Vitis C, Malanga D, Roscilli G, Noto A, Marra E, Laudanna C, Zoppoli P, De Luca P, Affuso A, Ruco L, Di Napoli A (2011). Spheres derived from lung adenocarcinoma pleural effusions: molecular characterization and tumor engraftment. PLoS One.

[10] Szulkin A, Otvos R, Hillerdal CO, Celep A, Yousef-Fadhel E, Skribek H, Hjerpe A, Szekely L, Dobra K (2014). Characterization and drug sensitivity profiling of primary malignant mesothelioma cells from pleural effusions. BMC Cancer.

[11] Szulkin A, Nilsonne G, Mundt F, Wasik AM, Souri P, Hjerpe A, Dobra K (2013). Variation in drug sensitivity of malignant mesothelioma cell lines with substantial effects of selenite and bortezomib, highlights need for individualized therapy. PLoS One.

[12] Boulikas T, Vougiouka M (2004). Recent clinical trials using cisplatin, carboplatin and their combination chemotherapy drugs (review). Oncol Rep.

[13] Sweeney CJ, Sandler AB (1998). Treatment of advanced (stages III and IV) non-small-cell lung cancer. Curr Probl Cancer.

[14] Shimizu J, Horio Y, Osada H, Hida T, Hasegawa Y, Shimokata K, Takahashi T, Sekido Y, Yatabe Y (2008). mRNA expression of RRM1, ERCC1 and ERCC2 is not associated with chemosensitivity to cisplatin, carboplatin and gemcitabine in human lung cancer cell lines. Respirology.

[15] Olaussen KA, Dunant A, Fouret P, Brambilla E, Andre F, Haddad V, Taranchon E, Filipits M, Pirker R, Popper HH, Stahel R, Sabatier L, Pignon JP (2006). DNA repair by ERCC1 in non-small-cell lung cancer and cisplatin-based adjuvant chemotherapy. N Engl J Med.

[16] Tantraworasin A, Saeteng S, Lertprasertsuke N, Arayawudhikul N, Kasemsarn C, Patumanond J (2013). The prognostic value of ERCC1 and RRM1 gene expression in completely resected non-small cell lung cancer: tumor recurrence and overall survival. Cancer Manag Res.

[17] Bepler G, Williams C, Schell MJ, Chen W, Zheng Z, Simon G, Gadgeel S, Zhao X, Schreiber F, Brahmer J, Chiappori A, Tanvetyanon T, Pinder-Schenck M (2013). Randomized international phase III trial of ERCC1 and RRM1 expression-based chemotherapy versus gemcitabine/carboplatin in advanced non-small-cell lung cancer. J Clin Oncol.

[18] Bepler G, Kusmartseva I, Sharma S, Gautam A, Cantor A, Sharma A, Simon G (2006). RRM1 modulated in vitro and in vivo efficacy of gemcitabine and platinum in non-small-cell lung cancer. J Clin Oncol.

[19] Olaussen KA, Soria JC (2010). Validation of ERCC1-XPF immunodetection--letter. Cancer Res.

[20] Twentyman PR, Wright KA, Mistry P, Kelland LR, Murrer BA (1992). Sensitivity to novel platinum compounds of panels of human lung cancer cell lines with acquired and inherent resistance to cisplatin. Cancer Res.

[21] Reynolds C, Obasaju C, Schell MJ, Li X, Zheng Z, Boulware D, Caton JR, Demarco LC, O'Rourke MA, Shaw Wright G, Boehm KA, Asmar L, Bromund J (2009). Randomized phase III trial of gemcitabine-based chemotherapy with in situ RRM1 and ERCC1 protein levels for response prediction in non-small-cell lung cancer. J Clin Oncol.

[22] Vilmar AC, Santoni-Rugiu E, Sorensen JB (2013). Predictive impact of RRM1 protein expression on vinorelbine efficacy in NSCLC patients randomly assigned in a chemotherapy phase III trial. Ann Oncol.

[23] Zhang Q, Zhu X, Zhang L, Sun S, Huang J, Lin Y (2014). A prospective study of biomarker-guided chemotherapy in patients with non-small cell lung cancer. Cancer Chemother Pharmacol.

[24] Valsecchi ME, Holdbrook T, Leiby BE, Pequignot E, Littman SJ, Yeo CJ, Brody JR, Witkiewicz AK (2012). Is there a role for the quantification of RRM1 and ERCC1 expression in pancreatic ductal adenocarcinoma?. BMC Cancer.

[25] Akita H, Zheng Z, Takeda Y, Kim C, Kittaka N, Kobayashi S, Marubashi S, Takemasa I, Nagano H, Dono K, Nakamori S, Monden M, Mori M (2009). Significance of RRM1 and ERCC1 expression in resectable pancreatic adenocarcinoma. Oncogene.

[26] Bhagwat NR, Roginskaya VY, Acquafondata MB, Dhir R, Wood RD, Niedernhofer LJ (2009). Immunodetection of DNA repair endonuclease ERCC1-XPF in human tissue. Cancer Res.

[27] Smith DH, Fiehn AM, Fogh L, Christensen IJ, Hansen TP, Stenvang J, Nielsen HJ, Nielsen KV, Hasselby JP, Brunner N, Jenssen SS (2014). Measuring ERCC1 protein expression in cancer specimens: validation of a novel antibody. Sci Rep.

[28] Otvos R, Skribek H, Kis LL, Gloghini A, Markasz L, Flaberg E, Eksborg S, Konya J, Gergely L, Carbone A, Szekely L (2011). Drug sensitivity patterns of HHV8 carrying body cavity lymphoma cell lines. BMC Cancer.

[29] Markasz L, Kis LL, Stuber G, Flaberg E, Otvos R, Eksborg S, Skribek H, Olah E, Szekely L (2007). Hodgkin-lymphoma-derived cells show high sensitivity to dactinomycin and paclitaxel. Leuk Lymphoma.

[30] Skribek H, Otvos R, Flaberg E, Nagy N, Markasz L, Eksborg S, Masszi T, Kozma A, Adam E, Miseta A, Klein E, Szekely L (2010). Chronic lymphoid leukemia cells are highly sensitive to the combination of prednisolone and daunorubicin, but much less to doxorubicin or epirubicin. Exp Hematol.

