# Supplementary material for: Drug sensitivity profiling and molecular characteristics of cells from pleural effusions of patients with lung adenocarcinoma
Source: Genes Cancer. 2015 Mar;6(3-4):119–28. doi: 10.18632/genesandcancer.56 (PMC4426949; doi:10.18632/genesandcancer.56)
Supplement: Supplementary file 1 [file ganc-06-119-s001.pdf]

Drug sensitivity profiling and molecular characteristics of cells from pleural effusions of patients with lung adenocarcinoma

Additional file 1: Chemotherapic agents used in cytotoxicity assay.

			In vitro concentrations (µg/ml)
**Alkylating Agents**	Nitrogen mustard	Chlorambucil	83.3
	Aziridine	Mitomycin C	0.3 - 0.6
	Tetrazine	Dacarbazine	16.7
**Platinum drugs**		Cisplatin	0.2
		Carboplatin	1.7
		Oxaliplatin	4.2
**Antimetabolites**	Pyrimidine analogues	Fluorouracil	41.7
		Cytarabine	8.3 - 16.7
		Gemcitabine	33.3
	Purine analogues	Mercaptopurine	69.4
		Fludarabine	20.8 - 41.7
		Cladribine	0.8
	Antifolates	Methotrexate	4.2 - 20.8
		Pemetrexed	20.8
	Other	Hydroxyurea	41.7
**Antimicrotubule agents**	Taxanes	Paclitaxel	3.3
		Docetaxel	8.3
	Vinca alkaloids	Vinblastine	0.8 - 8.3
		Vincristine	0.2 - 0.8
		Vinorelbine	1.7 - 8.3
**Topoisomerase inhibitors**	Type I	Topotecan	0.2 -1.7
		Irinotecan	16.7
	Type II	Etoposide	16.7
		Amsacrine	4.2
**Proteasome inhibitor**		Bortezomib	2.9
**Multifunctional drugs**	Anthracycline	Daunorubicin	4.2 - 16.7
		Doxorubicin	0.8 - 1.7
		Epirubicin	1.7
	Other	Actinomycin D	0.1 - 0.4
		Bleomycin	2.5 - 12.5*
**Enzyme**		Asparaginase	0.6 - 8.3*
**Corticosteroid**		Prednisolone	16.7 - 20.8

Drugs are divided according to their mechanism of action. * = IU/ml.

Additional file 2: Antibodies used in these experiments.

Target	Abbreviation	Antibody	Dilution	Supplier	Product code
Epithelial cell adhesion molecule	EpCAM	Mouse monoclonal	1:1000	2	M0804
Calretinin		Mouse monoclonal	1:100	2	M7245
Thyroid transcription factor-1	TTF-1	Mouse monoclonal	1:100	2	M3575
Napsin A		Mouse monoclonal	1:200	1	NCL-L-Napsin A
Leukocyte common antigen	CD45	Mouse monoclonal	1:400	1	NCL-LCA
Excision repair cross-complementation group 1	ERCC1	Mouse monoclonal Ab-2 (clone 8F1)	1:200	3	MS-671
Ribonucleotide reductase M1	RRM1	Rabbit polyclonal	1:50	4	Ab81085

Suppliers: 1 = Leica Microsystems GmbH, Wetzlar, 2 = Dako, Glostrup, Denmark, 3 = Thermo Fisher Scientific Inc, Waltham, MA, USA. 4 = Abcam, Cambridge, UK.

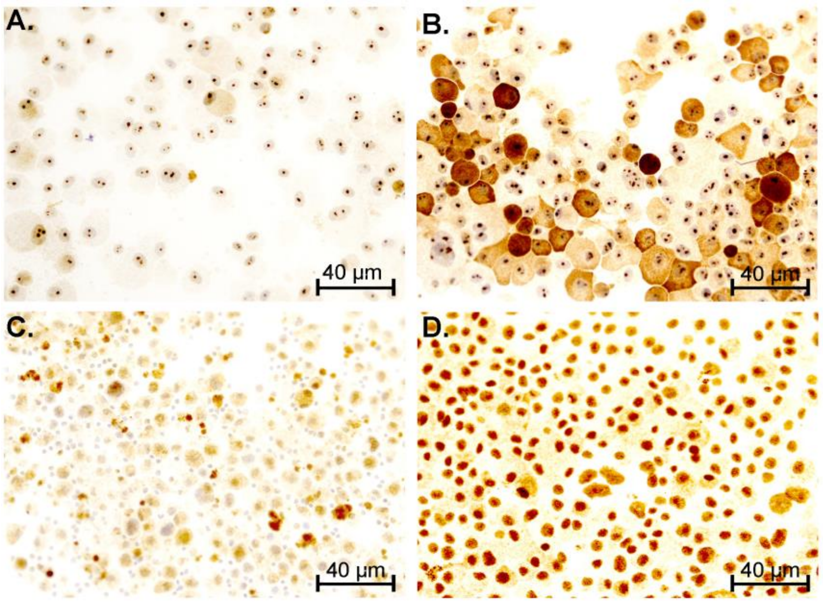

Additional file 3: RRM1 and ERCC1 immunoreactivity of lung adenocarcinoma cells. Representative micrographs presenting the RRM1 and ERCC1 immunocytochemical staining's. A: weak RRM1 staining intensity (score 1), B: strong RRM1 staining intensity (score 3), C: weak ERCC1 staining intensity (score 1) and D: strong ERCC1 staining intensity (score 3), respectively. Scale bar = 100 µm.
